# Assessing Whether Providing Regular, Free HIV Self-Testing Kits Reduces the Time to HIV Diagnosis: An Internet-Based, Randomized Controlled Trial in Men Who Have Sex With Men

**DOI:** 10.1097/QAI.0000000000003564

**Published:** 2025-02-05

**Authors:** David T. Dunn, Leanne McCabe, Denise Ward, Andrew N. Phillips, Fiona C. Lampe, Fiona Burns, Valerie Delpech, Peter Weatherburn, T. Charles Witzel, Roger Pebody, Peter Kirwan, Jameel Khawam, Sara Croxford, Michael Brady, Kevin A. Fenton, Roy Trevelion, Yolanda Collaco-Moraes, Sheena McCormack, Alison J. Rodger

**Affiliations:** a MRC Clinical Trials Unit at UCL, London, United Kingdom;; b Institute for Global Health, UCL, London, United Kingdom;; c National Infection Service, UK Health Security Agency, London, United Kingdom;; d Department of Public Health, Environments & Society, London School of Hygiene & Tropical Medicine, London, United Kingdom;; e AIDSmap GB, London, United Kingdom;; f Department of Sexual Health and HIV, King's College Hospital, London, United Kingdom;; g Department of Health and Social Care, London, United Kingdom; and; h HIV i-Base, London, United Kingdom.

**Keywords:** HIV, self-testing, diagnosis, internet-based, randomized

## Abstract

**Background:**

The risk of onward HIV transmission is strongly influenced by the interval between HIV infection and its diagnosis. The SELPHI trial examined whether this interval could be reduced by offering free HIV self-testing kits to men who have sex with men (MSM).

**Setting:**

Internet-based RCT of MSM aged ≥16 years, resident in England/Wales, recruited through sexual and social networking sites.

**Methods:**

The second-stage randomization of SELPHI was open to participants who used an initial free HIV self-test kit, were HIV seronegative, and reported recent condomless anal sex. They were randomized to receive a free HIV self-test kit every 3 months (repeat testing [RT] group) versus no such offer (nRT group). The primary outcome was time from randomization to a confirmed HIV diagnosis, determined from linkage to national HIV surveillance databases. The key secondary outcome was the frequency of HIV testing regardless of test modality.

**Results:**

In total, 2308 eligible participants (1161 RT, 1147 nRT) were randomized between April 2017 and June 2018, and followed for 15–27 months. The proportion of participants reporting an HIV test in the previous 3 months was much higher in the RT group (86%) than in the nRT group (39%). Overall, 16 (9 RT, 7 nRT) confirmed HIV diagnoses were observed (0.35/100 person-years), with no difference between the groups (hazard ratio = 1.27 [95% CI: 0.47 to 3.41], *P* = 0.63).

**Conclusions:**

Providing regular free self-testing kits to sexually active MSM was highly acceptable and markedly increased HIV testing. However, in this low incidence cohort, it did not result in a demonstrably more rapid diagnosis of incident infections.

## Abstract 2

Supplemental Digital Content is Available in the Text.

## INTRODUCTION

The prompt diagnosis of HIV infection and early initiation of antiretroviral therapy are clinically beneficial for the individual and confer population-level effect by suppressing HIV viral replication and eliminating the risk of onward HIV transmission.^1–3^ Modeling studies have shown that increasing the coverage and rate of HIV testing would significantly alter the course of the HIV epidemic among men who have sex with men (MSM).^4–7^ UK and international testing guidelines recommend 3-monthly testing for MSM at high risk of acquiring HIV infection,^8^ but we previously reported a low rate of compliance with this recommendation.^9^

Key barriers to HIV testing are difficulties in accessing sexual health services and stigma around the disclosure of sexual practices and sexual activity.^10^ HIV self-testing may circumvent these barriers and has been found to be acceptable in a variety of populations, particularly if users' values and preferences are taken into account in intervention development.^11,12^ However, most of the quantitative research on HIV self-testing has focused on its role in identifying prevalent, possibly long-standing, infections.^13^

SELPHI was a large, internet-based trial, designed to evaluate the impact of offering free HIV self-testing kits to MSM, with a 2-stage randomization. The first randomization, which has been published, found no demonstrable effect on the detection of undiagnosed, prevalent infections.^14^ In this study, we describe the results of the second randomization that assessed whether providing regular self-testing kits to HIV-seronegative MSM, deemed to be at higher risk of acquiring HIV, reduced the time to the clinically confirmed diagnosis of the infection.

## METHODS

### Study Design

**FIGURE 1.**
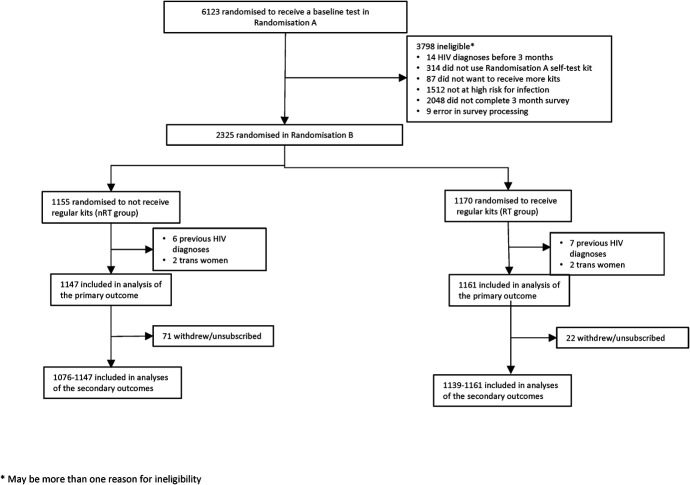
CONSORT diagram.

SELPHI (HIV Self-testing Public Health Intervention) was an open-label, internet-based randomized controlled trial with a 2-stage randomization (see Figure 1, Supplemental Digital Content, http://links.lww.com/QAI/C391), which has been described in full previously.^15^ In the first-stage randomization, which took place at enrollment, eligible participants were randomly allocated to the offer of a single free HIV self-test kit (BT group) or no such offer (nBT group).^14^ The second-stage randomization took place after the month 3 survey and was open to participants allocated to the BT group and who met further eligibility criteria. Participants were randomly allocated (1:1 ratio) to the offer of an HIV self-test kit every 3 months (repeat testing (RT) group) versus no such offer (nRT).

Participants to SELPHI were recruited through various online sexual and social networking sites. Eligibility criteria for both randomizations were age ≥16 years, resident in England/Wales, male or transgender woman, ever had anal sex with a man, not known to be HIV positive, and providing consent to link to the UK national HIV surveillance databases. Very few transgender women were recruited and their data have been described separately.^16^ Additional eligibility criteria for the second-stage randomization were responded to the month 3 survey, had used the self-test kit sent at enrollment, remained HIV negative, reported condomless anal sex (CAS) with ≥1 male partners since enrollment, and interested in using HIV self-test kits in the future.

All participants randomized to the second stage of the trial were sent a link every 3 months to complete follow-up surveys, which asked questions about sexual behavior and testing for HIV and for other sexually transmitted infections (STIs) in the previous 3 months, and current use of pre-exposure prophylaxis (PrEP). Follow-up was planned to continue until the last participant randomized had been followed for 2 years, although actual follow-up was shorter than this because of the end in study funding.

Survey invitations and responses were securely managed by Demographix, an online survey company. Extensive formative work was conducted before the trial to explore the acceptability of HIV self-testing among MSM and assess preferences for types of HIV self-testing kits.^17^ The kit selected for the trial, currently marketed as Chembio Sure Check, incorporates a second-generation antibody immunoassay detecting HIV-1/2 based on a whole blood sample from a finger prick (manufacturer's specifications: window period from infection = 28 days, sensitivity = 99.7%, specificity = 99.9%). The kits were posted directly to the participants by the manufacturer. Information encouraging testing for other STIs and signposting to HIV/sexual health websites was included in the kit.

### Outcomes

The primary outcome was the time from randomization (second-stage) to a clinically confirmed HIV diagnosis (this interval should be shorter if the intervention was effective). Ideally, we would have compared the groups in terms of the interval between the time of acquisition of HIV infection and its diagnosis, but the former is not generally observed; the primary outcome is, therefore, a surrogate for this measure. We note that the analysis would be invalid if the intervention affected the underlying HIV incidence rate, but we found no difference between the randomized groups in terms of STI diagnoses and the number of CAS partners, both markers of the risk of HIV infection (see Results).

Data on HIV diagnoses were primarily obtained from linkage to the national HIV surveillance databases, which are maintained by UK Health Security Agency (UKHSA).^18^ An attempt was made to contact participants who reported a positive HIV test (or a reactive self-test) but who did not link with the UKHSA databases to resolve the reason for the discrepancy. Only UKHSA diagnoses or verified self-reports were included. Participants who withdrew from completing the follow-up surveys are included in the UKHSA linkage and thus in the analysis of the primary outcome measure. The key secondary outcomes, as assessed from responses to the regular follow-up surveys, were the frequency of HIV testing (irrespective of testing modality), the frequency of STI screening, STI diagnoses, and the number of CAS partners.

### Sample Size and Statistical Analysis

The sample size of 10,000 for the overall trial was determined by the number of self-test kits that could be acquired within the study budget. It was assumed that 50% of the approximately 6000 participants enrolled in the BT group would meet the additional eligibility criteria and thus enter the second-stage randomization, that is, a total of 3000 (1500 in each of the RT and nRT groups). We used simulation to estimate the statistical power for a range of plausible parameter values.^15^ The estimated power, at 2-sided 5% significance level, for comparing the randomized groups with respect to the primary outcome, varied between 56% and 99% depending on the assumptions.^15^

Analyses of the primary outcome were performed using the intention-to-treat principle, regardless of the actual usage of HIV self-test kits by participants in the RT group. The analysis used time-to-event methods (Kaplan–Meier plot, Cox proportional hazards model) with time 0 defined as the date of the second-stage randomization. Final matching by UKHSA was performed in November 2020; the censoring data for the analysis were defined as December 31, 2019 because all diagnoses before this date are likely to have been reported. Analyses of the secondary outcomes were restricted to participants who completed the follow-up surveys (see Discussion for possible biases). Several of the secondary analyses were repeated in the subgroup of participants who reported 2 or more CAS partners in the previous 3 months, that is, a marker of current higher risk of acquiring HIV infection and the need for more regular testing. Analyses were done with STATA (version 15.0). Because of the large sample size, more emphasis should be placed on confidence intervals than on *P*-values.^19^

## RESULTS

### Baseline Characteristics and Follow-Up

In total, 6123 participants were allocated to receive a single, baseline HIV self-test between February 2017 and March 2018 in the first-stage randomization (Fig. 1). Of these, 3798 were deemed ineligible for the second-stage randomization after completion of the month 3 survey; most ineligibilities were because of noncompletion of the month 3 survey or reporting no CAS since enrollment. Of the remaining 2325 participants, 1170 were allocated to receive regular 3 monthly HIV self-test kits if requested (RT group) and 1155 were allocated to not receive this offer (nRT group). In total, 13 participants were later discovered to have had a previous HIV diagnosis before trial entry (through UKHSA linkage),^18^ and 4 were transgender women, leaving 2308 participants (1161 RT, 1147 nRT) who contributed to the analysis of the primary outcome.

Baseline characteristics are given in Table 1. Median age was 34 years (IQR 27–44), 89% participants were White, 81% born in the United Kingdom, and 47% were university educated. Only 60% had tested for HIV in the 12 months before initial enrollment to SELPHI, and 11% had never had a test. Overall, 51% reported a single CAS partner between the first-stage and second-stage randomizations, 31% reported 2–3 partners, 13% reported 4–9 partners, and 4% reported 10 or more partners. In total, 6% of participants were currently taking PrEP and another 5% had taken PrEP previously.

**TABLE 1.** Baseline Characteristics

	RT (n = 1161)	nRT (n = 1147)	Total (n = 2308)
Median (IQR) age (y)	34 (27, 44)	34 (27, 44)	34 (27, 44)
Trans man, %	10 (1)	6 (1)	16 (1)
Born in the United Kingdom, %	929 (80)	934 (81)	1863 (81)
Highest education qualification, %			
University	539 (46)	553 (48)	1092 (47)
Higher education	130 (11)	126 (11)	256 (11)
School	448 (39)	414 (36)	862 (37)
None	24 (2)	20 (2)	44 (2)
Missing	13 (1)	18 (2)	31 (1)
Ethnicity, %			
White	1036 (89)	1016 (89)	2052 (89)
Asian	20 (2)	32 (3)	52 (2)
Black	21 (2)	19 (2)	40 (2)
Mixed	47 (4)	35 (3)	82 (4)
Other/Do not know/undisclosed	37 (3)	45 (4)	82 (4)
Time since last HIV test, mo, %			
<3	215 (19)	228 (20)	443 (19)
3–12	488 (42)	461 (40)	949 (41)
>12	319 (27)	320 (28)	639 (28)
Never	126 (11)	130 (11)	256 (11)
Missing	13 (1)	8 (1)	21 (1)
Number of CAS partners in previous 3 months, %*			
1	597 (51)	590 (51)	1187 (51)
2–3	369 (32)	344 (30)	713 (31)
4–9	153 (13)	152 (13)	305 (13)
10+	42 (4)	61 (5)	103 (4)
Pre-exposure prophylaxis, %			
Current	62 (5)	86 (7)	148 (6)
Previous	53 (5)	52 (5)	105 (5)
Never	1044 (90)	1009 (88)	2053 (89)
Missing	2 (<1)	0 (0)	2 (<1)

All participants had used SELPHI kit in previous 3 months, by definition.

* Characteristic at point of second-stage randomization; otherwise at point of first-stage randomization.

CAS, condomless anal sex.

Surveys were sent every 3 months until November 2019. The earliest recruited participants were sent 9 surveys (27 months after randomization) and the latest recruited participants were sent 5 surveys (15 months after randomization). Survey completion rates declined from 84% at the initial survey to 69% at 12 months and 47% at 24 months, with rates slightly higher in the RT group than in the nRT group (see Table 1, Supplemental Digital Content, http://links.lww.com/QAI/C391).

### HIV Testing

Among participants who completed the follow-up surveys in the RT group, the proportion who requested another SELPHI self-test kit was consistently between 85% and 90% (see Table 1, Supplemental Digital Content, http://links.lww.com/QAI/C391). The most common reasons for not requesting another test were the participant still having unused kits or a perception of no longer being at risk of acquiring HIV infection. When expressed relative to all eligible participants, this percentage declined from approximately 80% to 55% because of declining rates of survey completion. Overall, the participants reported having used the previously received kit in 94% of responses. Of the remaining responses, in 62% the participants stated that they planned to use the kit later on themselves, 22% had tested elsewhere, 11% had given the test to a friend, and 5% were classified as other. A total of 5085 SELPHI self-test kits were distributed to participants in the RT group, an average of 4.4 kits per participant.

**FIGURE 2.**
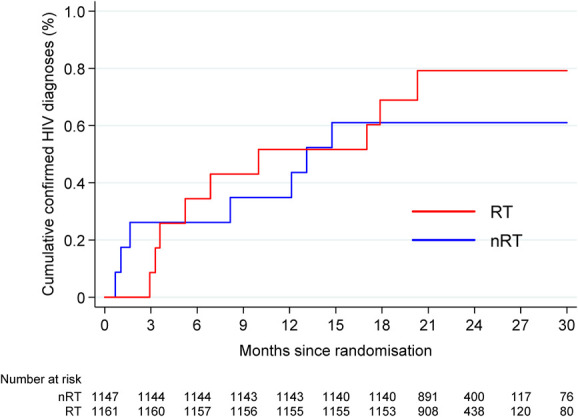
Cumulative proportion with a confirmed HIV diagnosis. Red line, RT group; blue line, nRT group.

Combining data across surveys, the proportion of participants reporting having had an HIV test (of any type) in the previous 3 months was considerably higher in the RT group (86%) than in the nRT group (39%), an absolute difference of 47% (95% CI: 44% to 50%) (Table 2, see Figure 2, Supplemental Digital Content, http://links.lww.com/QAI/C391). The respective values in an analysis limited to participants who reported ≥2 CAS partners in the previous 3 months were 92% and 53% (difference 38%, 95% CI: 35% to 42%). These percentages were stable across follow-up surveys, apart from participants in the nRT group reporting ≥2 CAS partners, in whom the rate of HIV testing increased during follow-up. Accordingly, the difference between the randomized groups narrowed at later surveys. This trend was almost entirely explained by an increase over time in the use of PrEP (see Results section).

**TABLE 2.** Proportion of Participants Reporting an HIV Test in the Previous 3 Months

Survey: months since Randomization	One or More HIV Tests in the Previous 3 mo					
	All Participants			≥2 CAS Partners in Previous 3 mo		
	nRT Group	RT Group	Difference* (95% CI)	nRT Group	RT Group	Difference* (95% CI)
3	322/953 (34%)	859/972 (88%)	55 (51 to 58)	178/401 (44%)	402/434 (93%)	48 (43 to 54)
6	312/862 (36%)	810/937 (86%)	43 (40 to 48)	148/268 (55%)	293/328 (89%)	34 (27 to 41)
9	333/810 (41%)	769/904 (85%)	46 (41 to 50)	123/213 (58%)	243/274 (89%)	31 (23 to 39)
12	304/729 (42%)	709/829 (86%)	44 (39 to 49)	91/157 (58%)	196/214 (92%)	34 (25 to 42)
15	273/675 (40%)	687/798 (86%)	44 (38 to 50)	87/135 (64%)	159/172 (92%)	28 (19 to 37)
18	224/544 (41%)	583/684 (85%)	44 (40 to 49)	67/103 (65%)	139/144 (97%)	31 (22 to 41)
21	135/329 (41%)	365/429 (85%)	44 (38 to 50)	41/54 (76%)	78/86 (91%)	15 (2 to 27)
24	51/115 (44%)	150/177 (85%)	40 (30 to 51)	13/16 (81%)	32/33 (97%)	16 (−4 to 36)
27	17/43 (40%)	46/55 (84%)	44 (27 to 62)	4/5 (80%)	11/11 (100%)	20 (−15 to 55)
Total	1971/5060 (39%)	4978/5785 (86%)	47 (44 to 50)	1187/2233 (53%)	2403/2622 (92%)	38 (35 to 42)

CI for total row allows for multiple observations per individual.

* Percentage in RT group minus percentage in nRT group.

In the RT group, having had an HIV test in the previous 3 months was reported on a total of 4975 occasions. Of these occasions, using a SELPHI kit only was reported 3250 (65%) times, a non-SELPHI test was reported 474 (10%) times, and the combined use of a SELPHI kit and another type of test was reported 1251 (25%) times. The use of a non-SELPHI test increased slightly, in relative terms, for the course of the surveys (see Figure 3, Supplemental Digital Content, http://links.lww.com/QAI/C391). The most common non-SELPHI tests were conducted in a sexual health clinic (1,063, 65%), a self-sample (tested in a laboratory) (240, 15%), or a self-test kit not provided by the study (194, 12%). In the nRT group, the most recent test was reported to be in a sexual health clinic (1094, 57%), a self-sample (416, 22%), a non-SELPHI self-test kit (240, 13%), or other (157, 8%).

### Primary Outcome: HIV Diagnosis

A total of 16 participants had a confirmed HIV diagnosis after randomization, 9 in the RT group and 7 in the nRT group (Fig. 2), with no evidence of a difference between the 2 groups (hazard ratio = 1.27 [95% CI: 0.47 to 3.41], *P* = 0.63). The overall incidence rate of new HIV diagnoses was 0.35 (95% CI: 0.22–0.58) per 100 person-years. One participant (nRT group) reported a positive HIV test that was not confirmed through UKHSA linkage; an attempt to contact this individual to confirm this result was unsuccessful.

Details of the 16 confirmed participants are given in Table 3, 11 of whom also self-reported their HIV diagnosis in a follow-up survey. Seven of the 9 participants in the RT group (exceptions H, I) reported having regularly used the SELPHI kit. Only 3 participants (L, M, P) reported a reactive self-test result, which presumably led to seeking a confirmatory test. This pathway is the most likely explanation for 3 other participants (K, N, O), although we cannot be certain because they did not complete the survey after their diagnosis, possibly because of the stress attendant on receiving an HIV diagnosis.

**TABLE 3.** Details of the Participants With a Confirmed HIV Diagnosis

Id	Month Infection diagnosed*	Diagnosis Self-Reported	Follow-Up Surveys Completed (mo)	Reported Tested for HIV in Previous 3 mo, by Survey	Reported STI (Non-HIV) Test in Previous 3 mo, by Survey	Reported CAS Partners in Previous 3 mo, by Survey
**nRT group**						
A	1	Y	3	Y	Y	4
B	1	Y	3	Y	Y	0
C	2	y	3	Y	Y	2
D	8	N	None	No data	No data	No data
E	12	Y	3, 6, 9, 12, 15	N,N,N,N,Y	N,N,N,N,Y	4, 4, 7–9, 5–6, 3
F	13	Y	3, 6, 9, 12, 15	N,N,N,N,Y	N,N,N,N,Y	1, 3, 4, 5–6, 1
G	15	Y	3, 6, 9, 12, 15, 18	N,Y,N,N,N,Y	N,N,N,N,N,Y	0, 0, 0, 1, 0, 2

Id	Month Infection Diagnosed*	Diagnosis Self-Reported	Follow-Up Surveys Completed (mo)	Reported Using Last Received SELPHI Kit, by Survey (Result)	Reported Non-SELPHI HIV Test in Previous 3 mo, by Survey	Reported STI (Non-HIV) Test in Previous 3 mo, by Survey	Reported CAS Partners in Previous 3 mo, by Survey
**RT group**							
H	3	Y	6	N	Y	Y	3
I	3	N	None	No data	No data	No data	No data
J	4	Y	3, 6	Y^-^,N	N,Y	N,Y	10+, 10+
K	5	N	3	Y^-^	Y	Y	0
L	7	Y	3, 6, 9	Y^-^,Y^-^,Y^+^	N,N,N	Y,N,Y	2, 0, 2
M	10	Y	3, 6, 9, 12	Y^-^,Y^-^,Y^-^,Y^+^	N,N,N,N	N,N,N,Y	0, 0, 0, 1
N	17	N	3, 6, 9, 12, 15	Y^-^,Y^-^,Y^-^,Y^-^,Y^-^	Y,N,N,N,N	Y,N,N,Y,Y	2, 2, 2, 3, 2
O	18	N	9, 12, 18	Y^-^,N,Y^-^	N,N,N	N,N,Y	5-6, 4, 5-6
P	20	Y	3, 6, 9, 12, 15, 18, 21	Y^-^,Y^-^,Y^-^,Y^-^,Y^-^,Y^-^,Y^+^	N,N,N,Y,N,N,N	N,N,N,N,N,N,Y	3, 4, 3, 7–9, 7–9, 4, 7-9

No participant reported using PrEP during follow-up other than participant N, who reported current PrEP usage at each survey.

* Time since randomization.

Of the 7 participants in the nRT group, 3 (A,B,C) had a confirmed diagnosis within 2 months of randomization; 1 participant (D), who was diagnosed at 8 months, did not complete any follow-up surveys; 3 participants (E,F,G) tested for HIV irregularly and it is unclear when they acquired HIV infection. Participants E and F reported multiple CAS partners at each survey, with the potential for onward viral transmission while the infection was undiagnosed. In contrast, participant G reported only 1 CAS partner (at month 12), implying that he likely acquired HIV between 9 and 12 months.

### STI Testing, STI Diagnoses, Sexual History, and PrEP

Combining data across surveys, the proportion of participants who reported a (non-HIV) STI test in the previous 3 months was marginally lower in the RT group (27%) than in the nRT group (29%, difference −2% [95% CI: −5% to 1%]) (see Table 2, Supplemental Digital Content, http://links.lww.com/QAI/C391). The difference was slightly more marked when the analysis was limited to participants who reported ≥2 CAS partners: 37% versus 41%, difference = −5% [95% CI: −9% to −1%]. Among participants who reported having had an STI test, there was no evidence of a difference between the randomized groups in the frequency of an STI diagnosis, most of which were either chlamydia or gonorrhea (see Tables 3, 4, Supplemental Digital Content, http://links.lww.com/QAI/C391). Consistent with this finding, there was no evidence that offering free HIV self-test kits affected sexual risk behavior as reflected by the number of CAS partners (see Table 5, Supplemental Digital Content, http://links.lww.com/QAI/C391). The use of PrEP increased markedly for the course of the study in both randomized groups, from 13% at the month 3 survey to 37% at the month 24 survey in all participants, and from 20% to 51% in participants who reported ≥2 CAS partners (see Table 6, Figure 4, Supplemental Digital Content, http://links.lww.com/QAI/C391). Of the 16 confirmed HIV participants, only 1 reported having used PrEP during follow-up (Table 3).

## DISCUSSION

The second-stage randomization of SELPHI trial found that providing regular free self-testing kits to sexually active MSM significantly increased the frequency of HIV testing but that this did not translate to a more rapid diagnosis of HIV infection and linkage to care. This mirrors the findings from the first-stage randomization, which found no benefit of providing a single self-testing kit in terms of identifying undiagnosed prevalent HIV infections.^14^ The overall incidence rate of diagnosed infections (0.35/100 person-years) was much lower than anticipated and the trial was consequently statistically underpowered.^15^ It is also considerably lower than the rate observed in the comparator (non-PrEP) group of the contemporaneous IMPACT study of PrEP rollout in England (0.95/100 person-years),^20^ indicating that SELPHI attracted men at intrinsically lower risk of HIV infection.^21^ Feasibility studies before SELPHI found that an important attraction of self-testing is the reassurance provided by an anticipated nonreactive result.^22^

The primary outcome in SELPHI—a laboratory-confirmed HIV diagnosis—was selected after careful deliberation. Other smaller trials have assessed HIV testing as the main outcome and, like us, demonstrated a positive effect.^13^ Although higher rates of testing would be predicted to lead to people with HIV being diagnosed earlier, we aimed to demonstrate this directly, given this is the key public health benefit. Although we found no statistical evidence of such an effect, there were at least 2 highly sexually active participants allocated to not receive HIV self-test kits whose HIV infection could have been diagnosed earlier and who may have transmitted the virus in the interim.

To date, only 1 other trial of HIV self-testing, which enrolled MSM in the United States, used newly identified HIV infections as a primary outcome.^23^ The investigators found a significantly higher rate of new HIV diagnoses in those allocated to self-testing, mainly because of the identification of prevalent rather than incident infections. However, all diagnoses were self-reported and survey completion rates were only approximately 60%, implying that many outcomes may have been missed. In SELPHI, linkage with the national HIV surveillance database ensured close to complete follow-up for the primary end point.^18^

SELPHI alleviated concerns about several perceived risks associated with HIV self-testing.^11,23^ First, the lack of face-to-face counseling before and after taking a test raises concerns that individuals may not seek linkage to care after a reactive self-test result. In SELPHI, only 3 out of 19 participants who reported a reactive result did not attend clinic for a confirmatory test (all baseline tests from the first-stage randomization, reported previously), with the possibility that the participants simply made an error on the online questionnaire.^14^ Our qualitative research found that those who had reactive self-tests looked to their social networks for support and linked to care promptly with encouragement from others.^24^ Second, by obviating the need to attend clinic, HIV self-testing could affect negatively on the rate of testing for other STIs. In SELPHI, there was a suggestion that the proportion reporting STI testing was lower in the RT arm, but the magnitude of this difference was small (2% overall). Were this to become an issue, consideration could be given to combining HIV self-testing with online testing for other STIs (currently, limited to self-sampling). Although there is increasing provision of online STI testing, the reliability of these tests has been questioned.^25^ Finally, although there is a theoretical risk that performing regular HIV self-tests could be associated with participation in more risky sex, we found no difference in the detection of STIs and the number of reported CAS partners.

It is important to note that the benefits of HIV self-testing extend beyond potentially improved diagnosis rates.^26^ Self-testing is empowering, placing individuals at the center of their own HIV testing process and decision making.^23,27^ It may also facilitate linkage to prevention interventions, such as PrEP, particularly among those estranged from clinical services.^28,29^ There are also unmeasured benefits if individuals redistribute self-testing kits to partners or friends. Finally, in settings where sexual health resources are constrained, HIV self-testing provides a low-cost testing option to complement existing services, freeing up capacity and clinical resources.^30^

The trial had several limitations. The participants were, by definition, interested in HIV self-testing and digitally literate, with an over-representation of White and highly educated men, and, therefore, not representative of all MSM nationally. The findings should be generalized cautiously and are most relevant in settings similar to the United Kingdom, with relatively good access to culturally competent sexual health services for MSM. Another limitation of the study is that sending regular surveys to participants may have reminded them to test for HIV, and preferentially biased the rates of testing in the nRT group. However, we still found a much higher rate of overall HIV testing in those allocated the trial intervention. Survey completion rates declined over time to approximately 50% in the nRT group and 60% in the RT group. This may have introduced bias into the analysis of the secondary outcomes given a plausible link between the likelihood of survey completion and positive health behaviors, such as testing for HIV and STIs. Finally, the duration of follow-up was insufficient to ensure that all new incident HIV infections had been diagnosed before the trial ended, that is, the trial selectively identified new infections with a comparatively short interval between infection and diagnosis.

PrEP became increasingly available in the United Kingdom during SELPHI, with around half of sexually active participants reporting its use by the end of follow-up in late 2019. This affected the results of the trial in 2 ways. First, its very high efficacy partly explains the lower than expected number of infections observed in the trial.^20,31^ Second, PrEP guidelines mandate 3-monthly HIV testing with a fourth-generation assay before further PrEP prescriptions are issued, which presumably increased the number of participants who were testing regularly.^32^ For PrEP users, there is no clear advantage testing for HIV in both clinics and self-testing, as was commonly observed in SELPHI. Although self-test kits are less accurate and have a longer window period, the very low risk of HIV infection among PrEP users suggests that self-testing could replace, at least in part, the need to attend a clinic for a test. Indeed, the HIV incidence rate in the United Kingdom is now much lower than the range of values considered in the modeling studies that demonstrated the cost-effectiveness of 3-monthly testing, on which current recommendations are based.^33,34^

In summary, our trial demonstrates that providing regular free HIV self-testing kits results in higher rates of testing, although it did not provide direct objective evidence of a more rapid diagnosis of incident cases of HIV. The data on HIV testing rates could potentially inform mathematical models on the population-level effect of prevention interventions on the number of future HIV infections and associated adverse clinical outcomes.^4–7^ More immediately, the high acceptability and use of the intervention, combined with a low, underlying HIV incidence rate, support a shift toward a more self-managed approach for HIV testing, where the individual primarily decides when testing is indicated.
